# Child malnutrition in sub-Saharan Africa: A meta-analysis of demographic and health surveys (2006-2016)

**DOI:** 10.1371/journal.pone.0177338

**Published:** 2017-05-11

**Authors:** Blessing J. Akombi, Kingsley E. Agho, Dafna Merom, Andre M. Renzaho, John J. Hall

**Affiliations:** 1 School of Science and Health, Western Sydney University, Penrith, New South Wales, Australia; 2 School of Social Sciences and Psychology, Western Sydney University, Penrith, New South Wales, Australia; 3 School of Public Health and Community Medicine, University of New South Wales, Sydney, New South Wales, Australia; Institut de recherche pour le developpement, FRANCE

## Abstract

**Background:**

Sub-Saharan Africa has one of the highest levels of child malnutrition globally. Therefore, a critical look at the distribution of malnutrition within its sub-regions is required to identify the worst affected areas. This study provides a meta-analysis of the prevalence of malnutrition indicators (stunting, wasting and underweight) within four sub-regions of sub-Saharan Africa.

**Methods:**

Cross-sectional data from the most recent Demographic and Health Surveys (2006–2016) of 32 countries in sub-Saharan Africa were used. The countries were grouped into four sub-regions (East Africa, West Africa, Southern Africa and Central Africa), and a meta-analysis was conducted to estimate the prevalence of each malnutrition indicator within each of the sub-regions. Significant heterogeneity was detected among the various surveys (I^2^ >50%), hence a random effect model was used, and sensitivity analysis was performed, to examine the effects of outliers. Stunting was defined as HAZ<-2; wasting as WHZ<-2 and underweight as WAZ<-2.

**Results:**

Stunting was highest in Burundi (57.7%) and Malawi (47.1%) in East Africa; Niger (43.9%), Mali (38.3%), Sierra Leone (37.9%) and Nigeria (36.8%) in West Africa; Democratic Republic of Congo (42.7%) and Chad (39.9%) in Central Africa. Wasting was highest in Niger (18.0%), Burkina Faso (15.50%) and Mali (12.7%) in West Africa; Comoros (11.1%) and Ethiopia (8.70%) in East Africa; Namibia (6.2%) in Southern Africa; Chad (13.0%) and Sao Tome & Principle (10.5%) in Central Africa. Underweight was highest in Burundi (28.8%) and Ethiopia (25.2%) in East Africa; Niger (36.4%), Nigeria (28.7%), Burkina Faso (25.7%), Mali (25.0%) in West Africa; and Chad (28.8%) in Central Africa.

**Conclusion:**

The prevalence of malnutrition was highest within countries in East Africa and West Africa compared to the WHO Millennium development goals target for 2015. Appropriate nutrition interventions need to be prioritised in East Africa and West Africa if sub-Saharan Africa is to meet the WHO global nutrition target of improving maternal, infant and young child nutrition by 2025.

## Introduction

Child undernutrition is a major public health problem, especially in many low-income and middle- income countries [1]. It adversely affects the productivity of nations as well as creating economic and social challenges among vulnerable groups. Poor nutrition is associated with suboptimal brain development, which negatively affects cognitive development, educational performance and economic productivity in adulthood [2].

Child growth is the most widely used measure of children’s nutritional status. The first 1000 days of life (0-23months) is a very critical phase in a child's life during which rapid physical and mental development occurs [3]. Undernutrition during this critical phase can have irreversible consequences on the child's growth leading to an increased risk of morbidity and mortality in children. Undernutrition is commonly assessed through the measurement of a child's anthropometry (height, weight), as well as through screening for biochemical and clinical markers [4]. Wasting, stunting and underweight are expressions of undernutrition and the anthropometric indicators for the assessment of a child’s nutritional status.

Undernutrition is the underlying cause of child mortality in about 45% of all deaths reported for children under-5 years of age [5]. In 2015, globally about 7.7% of children were wasted, 24.5% were stunted and 15% were underweight. The African region and South-East Asia have reported the highest prevalence of undernutrition, with the former accounting for about 39.4% of the stunted, 24.9% of the underweight and 10.3% of the wasted children under-5 years of age [6]. According to the 2015 Millennium development goal (MDG) report, sub-Saharan Africa (SSA) accounts for one third of all undernourished children globally, highlighting that malnutrition still remains a major health concern for children under 5 years in the sub-region, thus buttressing the need for urgent intervention [7].

There have been individual studies reporting the burden and determining factors of childhood undernutrition in SSA [8]. These individual studies have varied in design and geographic operationalisation, making it difficult to make regional comparisons and put in place regional initiatives to meet global agendas such as the MDGs. There have also been regional disparities in progress towards the MDG hunger and malnutrition targets, factors that contribute to these disparities are poorly understood. A pooled analysis can contribute towards addressing this gap and making informed regional comparisons. However, despite previous studies reporting the burden of child malnutrition across Africa, no study has critically analysed the pooled prevalence of each malnutrition indicator within the World Health Organization (WHO) geographical regions of SSA: West Africa, East Africa, Central Africa and Southern Africa. Hence, the aim of this study was to conduct a meta-analysis of malnutrition indicators in SSA using the most recent, nationally-representative Demographic and Health Surveys (DHS) (between 2006 and 2016) from 32 countries. Information from the pooled regional data will provide insight to the sub-regional distribution of undernutrition within SSA, thus assisting policy makers, global organizations, government and non-governmental organisations, the private sector and public health researchers in identifying the most vulnerable sub-regions within SSA where urgent nutrition related interventions are needed.

## Method

### Data sources

The data analysed in this study were extracted from the most recent Demographic and health survey (DHS) (2006–2016) of 32 SSA countries. The datasets are publicly available from the DHS website [9].

DHS collate data that are comparable across countries. The surveys are nationally representative and population-based with large sample sizes (usually between 5,000 and 30,000 households). Most surveys use a multi-stage cluster sampling method. Three core questionnaires are used in DHS surveys: a household questionnaire, a women’s questionnaire, and a men's questionnaire. In all households, women aged 15–49 years are eligible to participate; in many surveys men aged 15–59 years are also eligible to participate. Details on data collection and sampling methodology employed by DHS are described elsewhere [9].

#### Study selection and inclusion criteria

**Fig 1:**
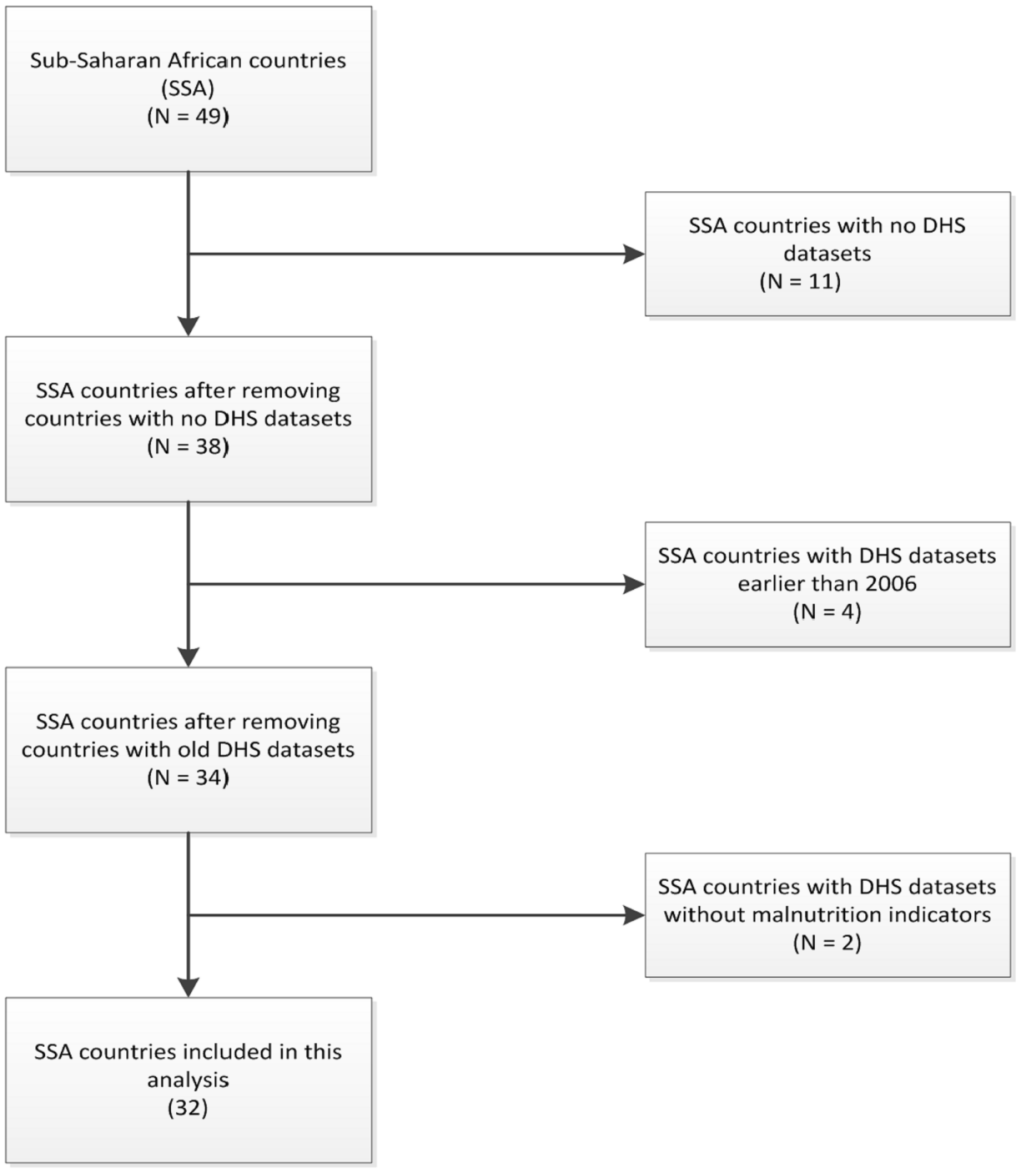
Flow chart for country selection.

Only countries with recent DHS (2006–2016) and comprehensive data on the anthropometric indicators of children under-5 years were included in this study. This inclusion of countries with DHS from 2006 to 2016 was so as to ensure the prevalence estimate is kept within a 10 year period given the high rates of malnutrition observed in the early 2000s following incidences of war and severe drought experienced in many countries prior to 2006. And also due to the introduction of the WHO child growth standards [10] in 2006, which estimated new values for the assessment of anthropometric indicators. Fig 1 shows the flow chart for country selection based on the inclusion criteria, while Table 1 presents the 32 countries included in this study, their respective sub-regions based on the United Nations (UN) geoscheme classification for SSA and the characteristics of their anthropometric indicators for children under-5 years.

**Table 1:** Countries of the different sub-regions in SSA and their anthropometric indicators for children under-5 years.

Country	Year of DHS	No of children	No of stunted children (%)	No of wasted children (%)	No of underweight children (%)
**East Africa**					
Burundi	2010	3590	2071 (57.7)	208 (5.8)	1034 (28.8)
Comoros	2012	2762	831 (30.1)	307 (11.1)	423 (15.3)
Ethiopia	2014	4921	1988 (40.4)	428 (8.7)	1240 (25.2)
Kenya	2014	18986	4936 (26.0)	759 (4.0)	2089 (11.0)
Malawi	2010	4849	2284 (47.1)	194 (4.0)	621 (12.8)
Mozambique	2011	10313	4393 (42.6)	609 (5.9)	1537 (14.9)
Rwanda	2015	3813	1445 (37.9)	84 (2.2)	355 (9.3)
Uganda	2011	2350	785 (33.4)	111 (4.7)	324 (13.8)
Tanzania	2016	9848	3348 (34.4)	1477 (4.5)	1379 (13.7)
Zambia	2014	12328	4944 (40.1)	740 (6.0)	183 (1.5)
Zimbabwe	2011	5260	1683 (32.0)	158 (3.0)	510 (9.7)
**West Africa**					
Burkina Faso	2010	6994	2420 (34.6)	1084 (15.5)	1798 (25.7)
Cote de Ivoire	2012	3581	1067 (29.8)	269 (7.5)	534 (14.9)
Gambia	2013	3372	826 (24.5)	388 (11.5)	546 (16.2)
Ghana	2014	2895	544 (18.8)	136 (4.7)	319 (11.0)
Guinea	2012	3531	1102 (31.2)	339 (9.6)	636 (18.0)
Liberia	2013	3520	1112 (31.6)	211 (5.9)	528 (15.0)
Mali	2013	4857	1860 (38.3)	617 (12.7)	1239 (25.0)
Niger	2012	5481	2406 (43.9)	987 (18.0)	1995 (36.4)
Nigeria	2013	26190	9638 (36.8)	2279 (8.7)	7517 (28.7)
Senegal	2011	3761	997 (26.5)	380 (10.1)	666 (17.7)
Sierra Leone	2013	5094	1931 (37.9)	474 (9.3)	835 (16.4)
Togo	2014	3282	903 (27.5)	213 (6.5)	525 (16.0)
**Southern Africa**					
Lesotho	2014	1869	617 (33.2)	56 (2.8)	187 (10.3)
Namibia	2013	2287	544 (23.8)	142 (6.2)	307 (13.4)
Swaziland	2007	2940	850 (28.9)	74 (2.5)	159 (5.4)
**Central Africa**					
Cameroun	2011	5860	1905 (32.5)	328 (5.6)	856 (14.6)
Chad	2014	10854	4330 (39.9)	1411 (13.0)	3125 (28.8)
Congo DR	2014	9030	3856 (42.7)	713 (7.9)	2041 (22.6)
Equatorial Guinea	2011	1094	287 (26.2)	34 (3.1)	61 (5.6)
Gabon	2012	3856	636 (16.5)	127 (3.3)	231 (5.9)
SaoTome&Principle	2009	1544	452 (29.3)	162 (10.5)	202 (13.1)

#### Malnutrition indicators

The malnutrition indicators analysed in this study are defined below:

Stunting *(Height-for-age)—*an indicator of linear growth retardation and cumulative growth deficits in children (Chronic malnutrition).
Wasting *(Weight-for-Height)*—measures body mass in relation to height and describes current nutritional status (acute malnutrition).
Underweight *(Weight-for-age)*—a composite index of height-for-age and weight-for-height. It takes into account both acute malnutrition (wasting) and chronic malnutrition (stunting), but it does not distinguish between the two.

In this study, data were collated for children with Z-score below minus two standard deviations (-2 SD) from the median of the WHO reference population [10]. Hence, stunting was defined as HAZ<-2; wasting as WHZ<-2 and underweight as WAZ<-2.

### Statistical analysis

This study was based on secondary data analysis. The syntax “metaprop” in Stata version 14.0 (StataCorp, College Station, TX, USA) [11] was used to generate forest plots for each of the malnutrition indicators. Each forest plot showed the prevalence of an indicator in individual countries and its corresponding weight, as well as the pooled prevalence in each sub-region and its associated 95% confidence intervals (CI). A test of heterogeneity of the DHS data obtained for the different countries showed a high level of inconsistency (I^2^ > 50%) thereby warranting the use of a random effect model in all the meta-analysis. Sensitivity analyses were conducted to examine the effect of outliers, by using a method similar to that employed by Patsopoulos and colleagues [12] which involves comparing the pooled prevalence before and after elimination of one country at a time.

## Results

The pooled prevalence of malnutrition for all 32 countries in the four sub-regions of SSA was 33.2% (95% CI: 30.4, 36.1) for stunting, 7.1% (95% CI: 6.0, 8.2) for wasting, and 16.3% (95% CI: 12.8, 19.9) for underweight.

### Stunting

**Fig 2:**
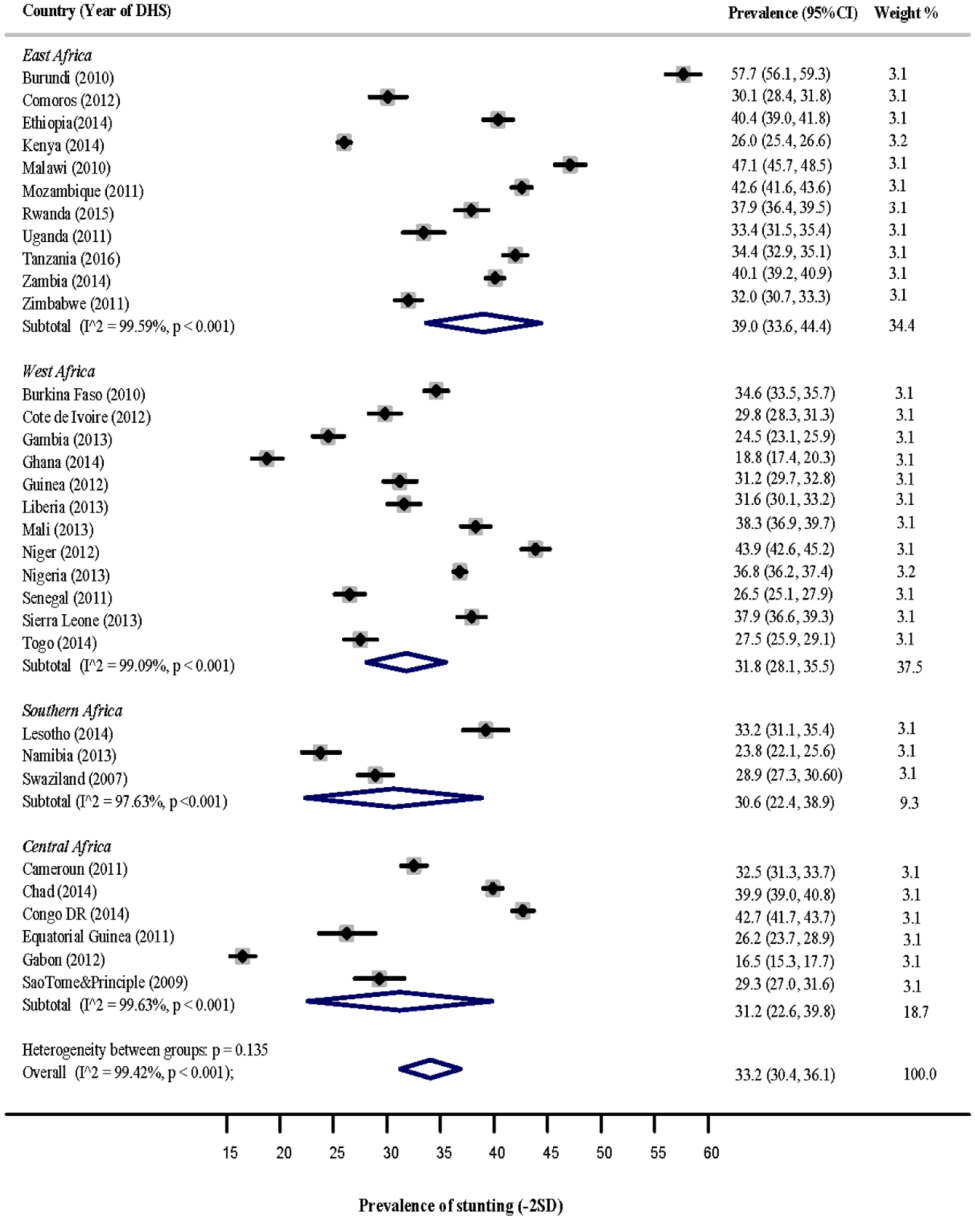
Prevalence of stunting among children under-5 years in the 4 sub-regions of SSA.

Fig 2 shows a forest plot of the prevalence of stunting among children under-5 years in the four sub-regions of SSA. The pooled estimate of stunting for East Africa was 39.0% (95% CI: 33.6, 44.4), West Africa [31.8% (95% CI: 28.1, 35.5)], Southern Africa [30.6% (95% CI: 22.4, 38.9)], and Central Africa [28.8% (95% CI: 21.4, 36.2)]. Countries with the highest significant prevalence of stunting in each sub-region were: Burundi [57.7% (95% CI: 56.1, 59.3)] and Malawi [47.1% (95% Cl: 45.7, 48.5)] in East Africa; Niger [43.9% (95% CI: 42.6, 45.2)], Mali [38.3% (95% Cl: 36.9, 39.7)], Sierra Leone [37.9% (95% Cl: 36.6, 39.3)] and Nigeria [36.8% (95% Cl: 36.2, 37.4)] in West Africa; Congo DR [42.7% (95% CI: 41.7, 43.7)] and Chad [39.9% (95% Cl: 39.0, 40.8)] in Central Africa. Lesotho reported the highest prevalence in Southern Africa [33.2% (95% CI: 31.1, 35.4)], however, this was not significant as its prevalence estimate overlapped with the pooled sub-regional estimate.

### Wasting

**Fig 3:**
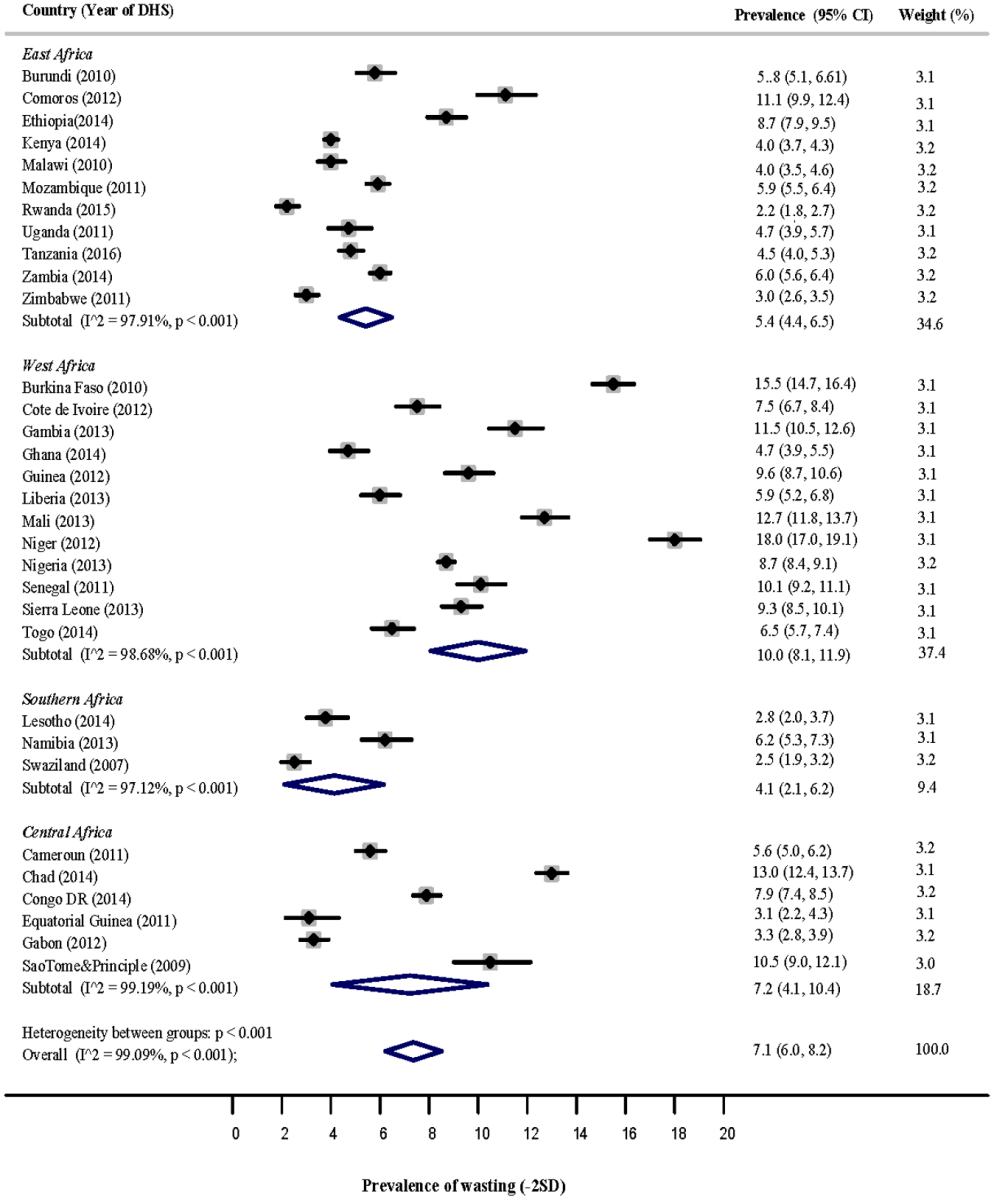
Prevalence of wasting among children under-5 years in the 4 sub-regions of SSA.

The forest plot displayed in Fig 3 shows the pooled estimate of wasting in the four sub-regions of SSA. The estimate for East Africa was 5.4% (95% CI: 4.4, 6.5), for West Africa 10% (95% CI: 8.1, 11.9), for Southern Africa 4.1% (95% CI: 2.1, 6.2), and for Central Africa 6.7% (95% CI: 4.2, 9.2). Countries that reported the highest significant prevalence of wasting in each sub-region were: Comoros [11.1% (95% CI: 9.9, 12.4)] and Ethiopia [8.70% (95% Cl: 7.9, 9.5)] in East Africa; Niger [18.0% (95% CI: 17.0, 19.1)], Burkina Faso [15.5% (95% Cl: 14.7, 16.4)] and Mali [12.7% (95% Cl: 11.8, 13.7)] in West Africa; Namibia [6.2% (95% CI: 5.3, 7.3)] in Southern Africa; Chad [13.0% (95% CI: 12.4, 13.7)] and Sao Tome & Principle [10.5% (95% Cl: 9.01, 12.1)] in Central Africa.

### Underweight

**Fig 4:**
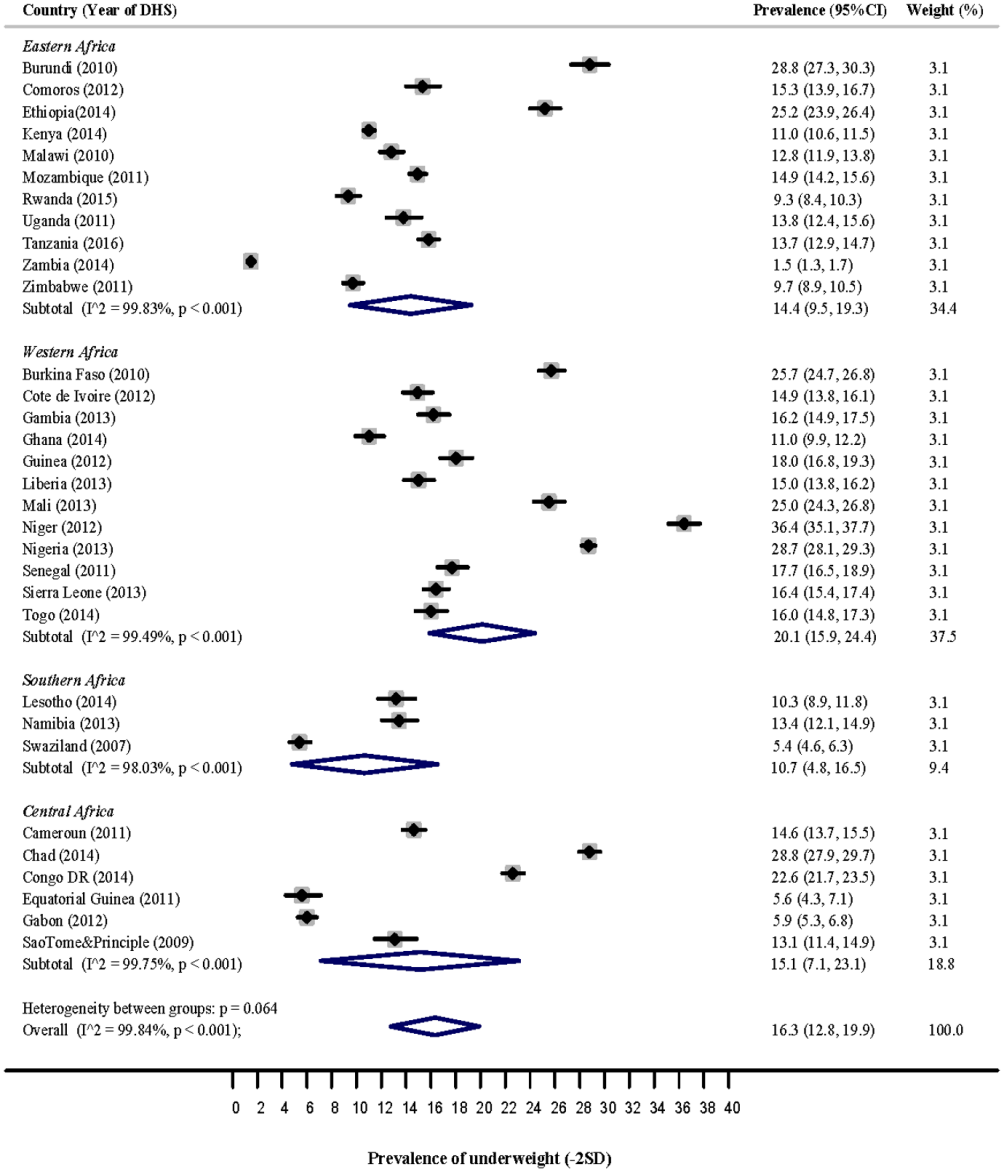
Prevalence of underweight among children under-5 years in the 4 sub-regions of SSA.

Fig 4 is a forest plot of the prevalence of underweight among children under 5 years in the four sub-regions of SSA. The pooled prevalence of underweight for East Africa was 14.4% (95% CI: 9.5, 19.6), for West Africa 20.1% (95% CI: 15.9, 24.4), for Southern Africa 10.7% (95% CI: 4.8, 16.5), and for Central Africa 12.8% (95% CI: 6.7, 18.9). Countries with the highest significant estimate of underweight in each sub-region were: Burundi [28.8% (95% CI: 27.3, 30.3)] and Ethiopia [25.2% (95% Cl: 23.9, 26.4)] in East Africa; Niger [36.4% (95% Cl: 35.1, 37.7)], Nigeria [28.7% (95% Cl: 28.1, 29.3)], Burkina Faso [25.7% (95% Cl: 24.7, 26.8)] and Mali [25.0% (95% Cl: 24.3, 26.8)] in West Africa; Chad [28.8% (95% Cl: 27.9, 29.7)] in Central Africa. Lesotho [13.2% (95%Cl: 11.8, 14.7) and Namibia [13.4% (95% CI: 12.1, 14.9)] reported the highest prevalence estimate in Southern Africa, however, this estimate was not significant as it overlapped with the pooled sub-regional estimate.

## Discussion

Exploring undernutrition on a regional basis often masks the differences in the burden of disease within sub-regions. This study investigated the sub-regional prevalence in undernutrition in children under five and reported stunting within countries in East Africa as the highest in the SSA region while wasting and underweight were highest within countries in West Africa. Burundi in East Africa had the greatest burden of disease for stunting while Niger in West Africa had the highest rates for wasting and underweight.

East Africa has the potential and capacity to produce enough food for its local consumption and a large surplus for export to the world market [13]. However, the region is grossly affected by food shortages, unfavourable climatic and drought conditions as well as limited access to land for agricultural purposes [14]. These factors seriously undermine progress toward improving agricultural productivity, food security and child nutrition in the region. Food insecurity has been known from previous studies to have an adverse effect on growth [15, 16, 17]; it could result from a lack of access to a stable food supply due to financial constraints or inadequate food exchange between places of abundant harvest and those with deficit due to poor transportation system, thus the need to ensure the four dimensions of food security and nutrition, i.e. availability, access, stability and utilization are effectively addressed [18]. In line with this, Leveraging Agriculture for Nutrition in East Africa (LANEA) mapped evidence across agriculture—nutrition pathways in East Africa and reported that effective food systems had positive impact child nutrition [19]. In India, Leveraging Agriculture for Nutrition in South Asia (LANSA) also reported that the agricultural sector contributes to dietary patterns of farm households and regulates food systems as well as income and expenditure which directly influence child nutrition [19]. Furthermore, the unfavourable climatic conditions experienced in East Africa could lead to high variability in food production and this could be caused by high variability of weather in the region which is likely to get worse due to climate change [18]. The frequency of droughts and floods has increased in East Africa over the past 30 years [14], thus affecting the region’s agricultural productivity.

Burundi reported the highest prevalence for stunting in the East Africa sub-region with nearly six in ten children under the age of five being stunted in 2010 [20]. The trend of stunting in Burundi has remained stable at 57.5% since it was first measured in 2005; without intervention, no improvements will be achieved and a further deterioration of the situation is inevitable [21].

To reduce stunting in the sub-region, nutrition policies and programmes prioritizing food security, improving agricultural productivity and implementing contingency actions in the face of climate change are urgently needed.

This study reported West Africa as having the highest rates for wasting and underweight in young children in SSA. West Africa has experienced very slow progress in reducing child undernutrition in the past 30 years [22]. The demographic, socio-economic and agro-ecological characteristics of the sub-region have adversely influenced the nutritional status of children as rapid population growth, rising cost of living and desertification has affected food access, availability and production especially within the arid Sahelian countries of West Africa [23]. The sub-region has recorded a population growth of 2.7% annually over the last thirty years and currently is estimated at over 300 million people. The population is expected to reach 388 million by 2020 and 490 million by 2030 [22]. However, the region's agriculture productivity has failed to keep up with population growth, thus resulting in high levels of food insecurity and poor nutritional outcome for children under five years [22], hence the need for educational programmes geared towards sustainable family growth to regulate population growth. This approach has been reported to lead to an effective reduction in population growth and family size, hence, contributing towards improving nutritional standards by increasing the available per capita food supply [24].

Niger reported the highest prevalence for both wasting and underweight in West Africa with nearly half of its children under 5 years being undernourished; this could be a result of the meagre farming and stockbreeding season for the year 2009–2010, when the country was hit by a serious food crisis [25] and has since struggled to meet its nutrition requirements. In order to reduce wasting and underweight in Niger in particular and West Africa in general, there is a need to diversify and increase food production as well as establishing a comprehensive food-based distribution strategy aimed at improving food security and child nutrition.

In general, the underlying causes of child malnutrition are similar across all countries in SSA and globally as outlined in the UNICEF conceptual framework for child nutrition [26]. However, poverty remains the principal cause of child undernutrition in SSA with 48.55% of the population living on less than $1.25 per day [27]. The rapid rise in world food prices and cost of living has transformed food insecurity from a difficult development problem into a nutrition crisis for underprivileged households [28]. The majority of the poor people reside in the rural areas and lack access to basic health services. The causes of poverty are related to harmful economic systems, conflict, environmental factors such as drought and climate change, and population growth [28]. Therefore, addressing poverty with a goal to improve food security and child nutrition is critical in strengthening livelihoods among vulnerable populations.

Our study has a number of strengths. First, the DHS are nationally representative and population-based with a large sample size. Second, the variables used by DHS are similarly defined in all surveys, thus comparable across all countries [9]. Third, our analysis was based on 32 countries with a recent DHS report. Hence, our study findings can be generalised to the entire sub-region. However, the study was limited as some SSA countries were not included because they had no recent DHS data (2006–2016) or had no comprehensive data on the malnutrition indicators. In addition, our study restricted the malnutrition indicators to stunting, wasting and underweight and did not include overweight.

### Policy implications

A multi-sectoral approach is vital in tackling undernutrition in SSA. This approach calls for holistic inter-organizational and inter-agency efforts to achieve food security, proper nutrition and poverty reduction over the long-term, and to improve agricultural capacity to meet demand against a backdrop of population growth and climatic variability. Policies that support cost-effective interventions around family planning and nutrition health education which takes into consideration the socio-cultural and environmental peculiarities of each sub-region, are urgently needed to reduce malnutrition in SSA.

Findings from this study will enable policy makers, global organizations, government and non-governmental organisations, the private sector as well as public health researchers to ascertain the most vulnerable sub-region in SSA for policy actions in the fight to reduce malnutrition in SSA.

## Conclusion

This study reveals the countries within sub-regions in SSA with the highest rates of stunting, wasting and underweight. Therefore, there is an urgency for strategic interventions aimed at improving child nutrition in the most vulnerable countries (Burundi, Malawi, Comoros, Ethiopia, Niger, Mali, Nigeria, Burkina Faso, Sierra Leone, Namibia, Chad and Congo DR) and sub-regions (East Africa and West Africa) in SSA if the internationally agreed WHO global nutrition targets regarding stunting, wasting and underweight among children under 5 years of age are to be achieved by 2025.

## Supporting information

S1 Checklist PRISMA 2009 checklist. (DOCX)
